# Leukemia inhibitory factor protects cholangiocarcinoma cells from drug-induced apoptosis via a PI3K/AKT-dependent Mcl-1 activation

**DOI:** 10.18632/oncotarget.4482

**Published:** 2015-07-20

**Authors:** Stuart Duncan Morton, Massimiliano Cadamuro, Simone Brivio, Marta Vismara, Tommaso Stecca, Marco Massani, Nicolò Bassi, Alberto Furlanetto, Ruth Elizabeth Joplin, Annarosa Floreani, Luca Fabris, Mario Strazzabosco

**Affiliations:** ^1^ Department of Molecular Medicine, University of Padua, Padua, Italy; ^2^ Department of Surgery & Translational Medicine, University of Milan-Bicocca, Milan, Italy; ^3^ Fourth Surgery Division, Treviso Regional Hospital, Treviso, Italy; ^4^ Department of Surgical, Oncological and Gastroenterological Sciences, University of Padua, Padua, Italy; ^5^ Pathology Unit, Treviso Regional Hospital, Treviso, Italy; ^6^ School of Immunity and Infection, University of Birmingham, Birmingham, UK; ^7^ Section of Digestive Diseases, Yale University School of Medicine, New Haven, CT, USA

**Keywords:** cholangiocarcinoma, leukemia inhibitory factor, chemoresistance, Mcl-1, phosphatidylinositol-3 kinase

## Abstract

Cholangiocarcinoma is an aggressive, strongly chemoresistant liver malignancy. Leukemia inhibitory factor (LIF), an IL-6 family cytokine, promotes progression of various carcinomas. To investigate the role of LIF in cholangiocarcinoma, we evaluated the expression of LIF and its receptor (LIFR) in human samples. LIF secretion and LIFR expression were assessed in established and primary human cholangiocarcinoma cell lines. In cholangiocarcinoma cells, we tested LIF effects on proliferation, invasion, stem cell-like phenotype, chemotherapy-induced apoptosis (gemcitabine+cisplatin), expression levels of pro-apoptotic (Bax) and anti-apoptotic (Mcl-1) proteins, with/without PI3K inhibition, and of pSTAT3, pERK1/2, pAKT. LIF effect on chemotherapy-induced apoptosis was evaluated after LIFR silencing and Mcl-1 inactivation.

Results show that LIF and LIFR expression were higher in neoplastic than in control cholangiocytes; LIF was also expressed by tumor stromal cells. LIF had no effects on cholangiocarcinoma cell proliferation, invasion, and stemness signatures, whilst it counteracted drug-induced apoptosis. Upon LIF stimulation, decreased apoptosis was associated with Mcl-1 and pAKT up-regulation and abolished by PI3K inhibition. LIFR silencing and Mcl-1 blockade restored drug-induced apoptosis.

In conclusion, autocrine and paracrine LIF signaling promote chemoresistance in cholangiocarcinoma by up-regulating Mcl-1 via a novel STAT3- and MAPK-independent, PI3K/AKT-dependent pathway. Targeting LIF signaling may increase CCA responsiveness to chemotherapy.

## INTRODUCTION

Cholangiocarcinoma (CCA) is a highly aggressive cancer arising from epithelial cells lining intrahepatic (iCCA) or extrahepatic (eCCA) bile ducts. Although considered a rare tumor, incidence of CCA (particularly iCCA) has steadily increased over the last few decades [1]. Despite this trend, treatment options remain limited to surgical resection and liver transplantation, and the overall survival beyond a year from diagnosis still remains less than 5% [1, 2]. In fact, resection can only be offered to a minority of patients (20–40%) because of a propensity for early intrahepatic or lymph node metastatic dissemination, whereas liver transplant is available only for carefully selected cases in a few, highly-specialized liver centers [2, 3]. Both procedures are further complicated by high rates of recurrence [2, 3]. For patients ineligible for surgery, palliation including radiotherapy, photodynamic therapy or stenting to relieve biliary obstruction, may provide some benefit [2, 4]. Notably, chemotherapy is recognized as largely ineffective due to the high resistance of CCA cells to drug cytotoxicity [5]. A recent study shows that combined administration of gemcitabine (GEM) and cisplatin (CDDP) in the treatment of advanced CCA increases patient overall survival [6] of about four months compared with patients treated with GEM alone [6].

Mechanisms of chemoresistance in CCA are poorly understood but the extensive desmoplastic reaction typical of CCA has been suggested to play a role. In fact, the close interplay between the cancer and surrounding stromal cells, i.e. cancer-associated fibroblasts (CAF) and tumor-associated macrophages (TAM), may be responsible for providing cancer cells with several pro-invasive functions, including proliferation, invasion, migration and resistance to apoptosis [7, 8]. Among the cytokines released within the tumor microenvironment, interleukin (IL)-6 plays a pivotal role in CCA pathogenesis, as a potent stimulator of cancer growth and progression [9, 10].

Leukemia inhibitory factor (LIF) is a pleiotropic pro-inflammatory cytokine belonging to the IL-6 superfamily secreted by a variety of cells including epithelial and stromal cells (fibroblasts, monocytes, macrophages and T-cells), albeit generally at very low levels [11]. However, LIF secretion may be stimulated by pro-inflammatory cytokines, such as IL-6, IL-1 and tumor necrosis factor (TNF)-α, leading to elevated serum LIF levels in cancer patients [12, 13]. LIF effects on cell functions are multifaceted, but still not extensively detailed. They include differentiation and maintenance of pluripotency, proliferation and apoptosis, pro- and anti-inflammatory stimuli, depending on the cell maturity and the cell type that the cytokine is acting upon [11, 13, 14]. Following LIF binding, the low affinity LIF receptor (LIFR) dimerizes with the glycoprotein (gp) 130 subunit, to form a high-affinity complex which transduces the LIF signal through different intracellular pathways, including Janus kinase (JAK)-signal transducer and activator of transcription (STAT), mitogen-activated protein kinase (MAPK)/extracellular signal-regulated kinase (ERK) or phosphatidylinositol-3 kinase (PI3K) [15]. Increasing numbers of studies support LIF as an important player in tumorigenesis and metastatic spread in various epithelial cancers [16–19]. LIF and LIFR/gp130 were found to be expressed in most of 30 human carcinoma cell lines [20]. However, LIF effects were extremely variable, and often opposing, as it induced proliferation in breast and pancreatic carcinomas but apoptosis in colon and gastric carcinomas; these effects were strictly influenced by the signaling pathways activated [20].

To date, very little is known about the role of LIF in CCA. Therefore, in this study, we sought: 1) the distribution of LIF, LIFR, and gp130 in human CCA liver tissue derived from surgical resection; 2) the secretion of LIF and expression of LIFR in primary and established human CCA cell lines; 3) the functional effects of LIF on CCA cells with respect to: a) cell proliferation and invasion, b) cell viability and apoptosis in response to the chemotherapeutic agents currently used in CCA (GEM, CDDP), c) the induction of a stem cell-like phenotype; d) the expression of pro- and anti-apoptotic proteins; and e) the downstream effectors of the signal transduction.

## RESULTS

### LIF, LIFR and gp130 were extensively expressed by bile ducts in CCA compared to peritumoral tissue

**Figure 1:**
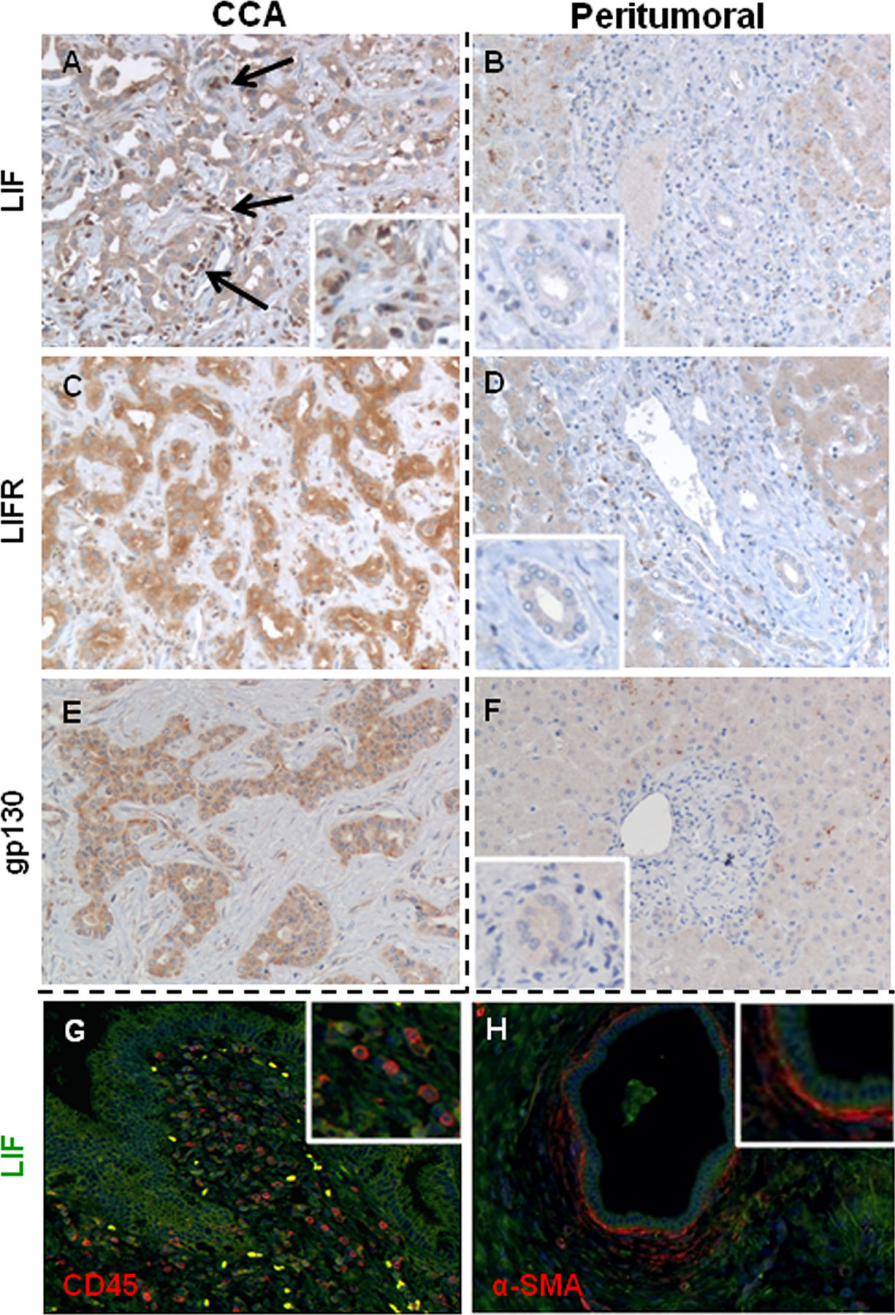
LIF, LIFR and gp130 immunohistochemical expression in CCA and peritumoral areas of human liver samples In CCA bile ducts, the extensiveness of LIF **A.** expression was heterogeneously distributed amongst samples, whilst the staining of LIFR **C.** and gp130 **E.** was more homogeneous. In contrast, LIF **B.**, LIFR **D.** and gp130 **F.** immunoreactivity was significantly less in bile ducts of matched peritumoral tissues. By immunohistochemistry and dual immunofluorescence we demonstrate that LIF (green) was also extensively expressed by CD45^+^ inflammatory cells (red, **G.**) and α-SMA^+^ cells (CAF, red, **H.**) that juxtaposed neoplastic biliary structures (A, black arrows and inset, G, and H) (Original magnification: A-H, 200 ×; insets, 400 ×).

Analysis of histological sections from resected human CCA liver revealed a significantly more extensive immunoreactivity of LIF (*p* < 0.001) and LIFR (*p* < 0.001) (Table 1) on bile ducts in tumoral areas (Figure 1A, 1C) compared with matched, peritumoral tissue (Figure 1B, 1D). Bile ducts of peritumoral areas were LIF-negative in all 12 samples, whilst 17/19 (89%) of neoplastic tissue contained LIF-positive bile ducts of different degree (Table 1). Similarly, the tumor reactive stroma surrounding the neoplastic bile ducts showed more extensive LIF immunoreactivity than the peribiliary stroma in peritumoral tissue (*p* < 0.001) (Table 1). Immunofluorescence studies revealed, more specifically, that in the tumor reactive stroma, LIF was expressed by inflammatory cells (CD45 positive), likely including macrophages, lymphocytes and neutrophils as evaluated by immunoperoxidase, and CAF (α-smooth muscle actin (α-SMA) positive) (Figure 1G, 1H). Only 4/12 peritumoral samples (33%) had extensive (>30%) LIFR staining in bile ducts, however, extensive LIFR positivity in neoplastic bile ducts was present in 17/19 (89%) CCA samples (Table 1). Gp130 expression on bile ducts in CCA and peritumoral tissue paralleled that of LIFR (Figure 1E, 1F). By categorizing the CCA areas, a significantly higher extent of LIF staining in ‘ductular-like’ than in ‘mucin-producing’ tumoral bile ducts was determined (Supplementary Figure 1A, 1C); in contrast, no significant differences in the extent of LIFR staining were found between the two CCA subtypes (Supplementary Figure 1B, 1C).

**Table 1:** Extent of LIF and LIFR-positive bile ducts/stromal cells in CCA and peritumoural areas of resected liver tissue sections (0 = <5%; 1 = 5–30%; 2 = 30–70%; 3 = >70% area of positive ducts)

LIF				
	BILE DUCTS		STROMAL CELLS	
Score	CCA	Peritumoral	CCA	Peritumoral
**0**	2	12	1	7
**1**	7	0	8	4
**2**	9	0	8	1
**3**	1	0	2	0
**Total**	19	12	19	12

LIFR		
	BILE DUCTS	
Score	CCA	Peritumoral
**0**	1	2
**1**	1	6
**2**	5	3
**3**	12	1
**Total**	19	12

### LIFR protein expression was greater in CCA than controls

**Figure 2:**
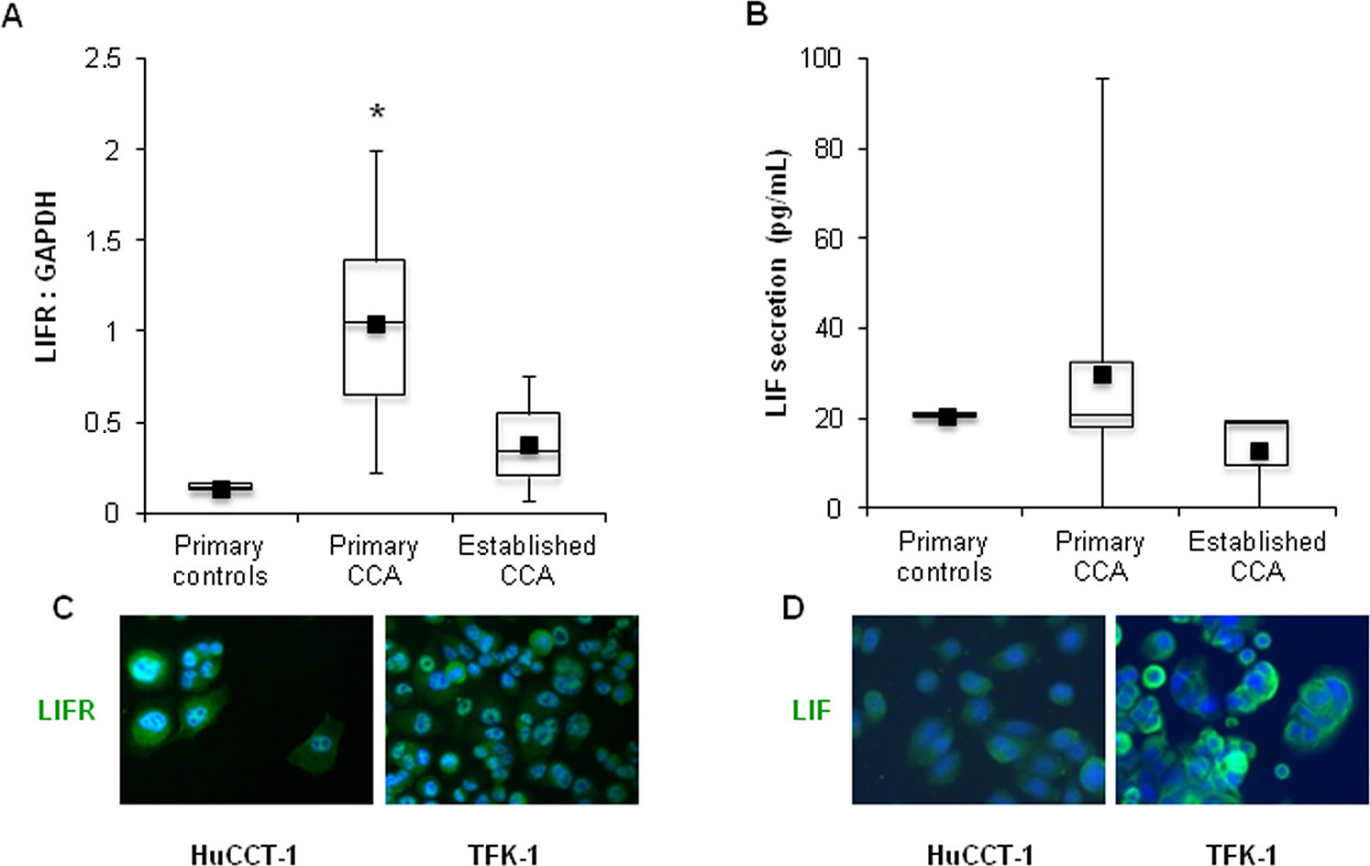
LIFR and LIF expression in human primary and established CCA cell lines By WB, LIFR protein levels were higher although variable in primary (*n* = 7) and established (*n* = 3) CCA cell lines compared with control (*n* = 2) cholangiocytes **A.** Using ELISA, LIF was found to be secreted by both neoplastic and control cholangiocytes, however with a large variability **B.** Of the established CCA cell lines, only HuCCT-1 and TFK-1 cells expressed LIFR **C.** and LIF **D.**, as shown by immunocytochemistry, which were then selected for *in vitro* experiments (Original magnification: 200x; *p < 0.05 vs. primary controls).

Relative amounts of LIFR protein obtained from primary and established CCA cell lines, and control cholangiocytes were evaluated by Western blotting (WB). Although LIFR protein expression levels were heterogeneous amongst CCA cholangiocytes, the average level was 7 times greater than that of the control (1.05 ± 0.56 vs. 0.14 ± 0.03) (Figure 2A).

### LIF secretion by cholangiocytes was variable

Using ELISA, no significant difference was found between the amount of LIF secreted by primary cholangiocytes from CCA and controls (29.9 ± 28.7 vs. 20.7 ± 0.3 pg/mL). However, the amount of LIF secreted by primary CCA cholangiocytes was extremely variable, ranging from 0 to 95.7 pg/mL (Figure 2B). Amongst the established CCA cell lines, HuCCT-1 (iCCA) and TFK-1 (eCCA) expressed LIFR and secreted LIF (Figure 2A, 2B), as confirmed by immunofluorescence in cultured cells (Figure 2C, 2D), therefore these cell lines were selected for subsequent *in vitro* experiments.

Data on LIFR expression and LIF secretion (obtained by WB analysis and ELISA respectively) were further confirmed by real-time PCR in established and primary CCA cell lines as well as in control cholangiocytes (Supplementary Figure 2A, 2B).

### LIF did not induce proliferation and invasion of established CCA cell lines, whilst it protected from apoptosis induced by chemotherapeutic agents

**Figure 3:**
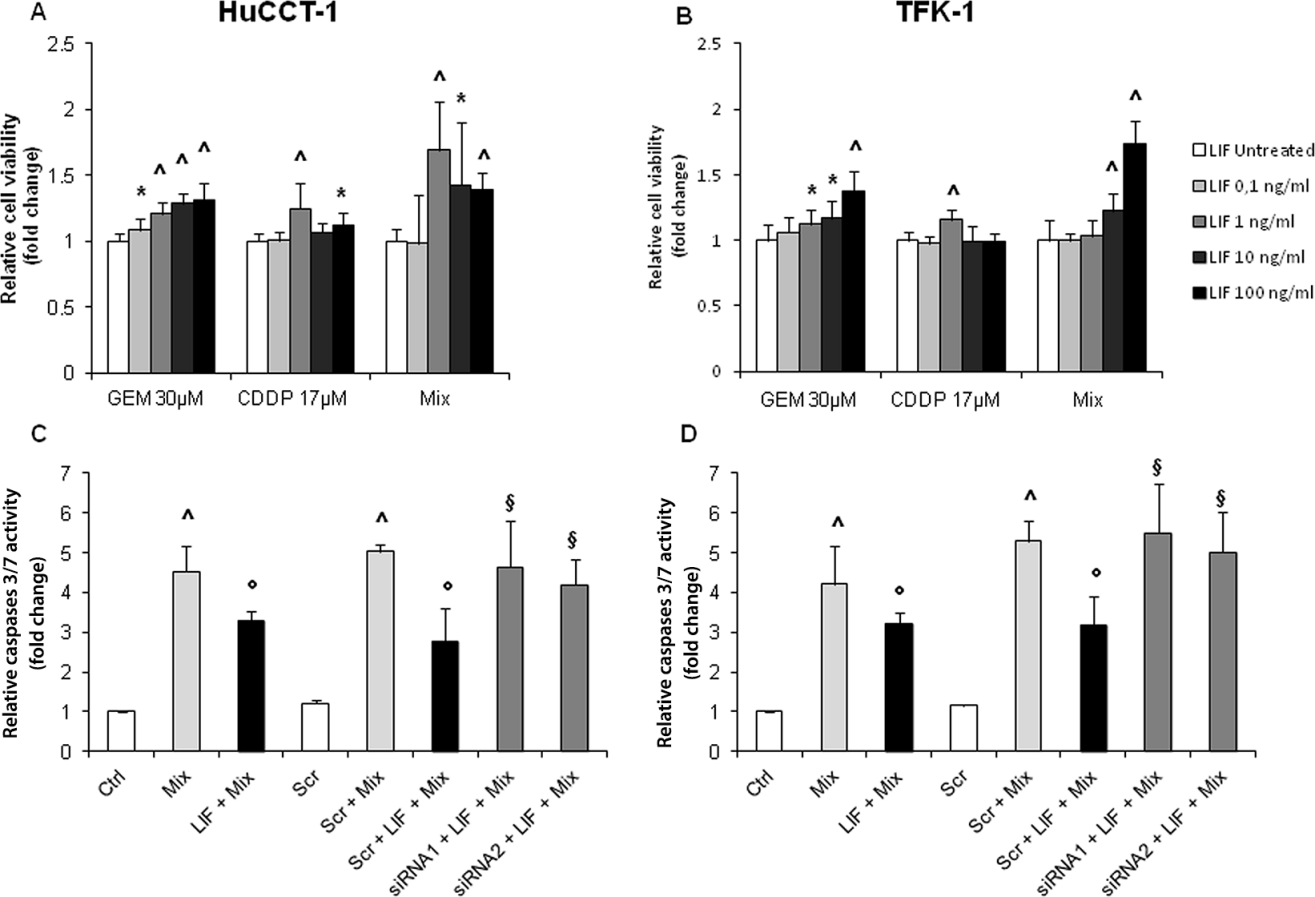
Protective effects of rhLIF on cell viability and apoptosis of CCA cells challenged with chemotherapeutic agents (GEM, CDDP, or GEM+CDDP) As evaluated by MTS assay, the pre-treatment of HuCCT-1 **A.** and TFK-1 **B.** cells with rhLIF was able to significantly counteract the cytotoxic effect of the chemotherapeutic agents, GEM, CDDP and GEM+CDDP (Mix). By caspase GLO 3/7 assay, the activation of caspases 3/7 in response to Mix in both CCA cell lines (**C, D** pale gray columns) was significantly reduced by the pre-treatment with rhLIF (100 ng/mL) (C, D black columns). Notably, the reduction in LIFR expression by two specific siRNAs was able to abolish the cytoprotective effects of rhLIF (C, D dark gray columns) (*p < 0.05 vs. untreated; ^*p* < 0.01 vs. untreated; °*p* < 0.05 vs Mix treatment; ^§^*p* < 0.05 vs Scr treated with Mix; *n* = minimum of 3 in duplicate).

HuCCT-1 and TFK-1 cells challenged with increasing doses of recombinant human (rh) LIF did not show any significant increase in the proliferative rate, except for a minimal change with the lowest dose in TFK-1 cells (Supplementary Figure 4A, 4B). Additionally, no change in invasive functions was observed with both CCA cell lines in response to LIF (Supplementary Figure 4E, 4F). To understand whether lack of LIF's proliferating effects was affected by autocrine LIF production by CCA cells, possibly inducing a constitutive activation of cell proliferation which precludes further activation upon ligand stimulation, we evaluated MTS assay in CCA cells with genetic inactivation of LIFR. The quality of the reduction in LIFR expression in HuCCT-1 and TFK-1 cells was evaluated by both real-time PCR and WB using 3 different siRNAs (Supplementary Figure 3). Using the 2 most effective siRNAs (siRNA1 and siRNA2), LIF's effects on cell proliferation were evaluated by comparing silenced cells with scrambled controls in absence of rhLIF stimulation. No MTS decrease was found in LIFR silenced cells compared with scrambled controls (Supplementary Figure 4C, 4D). We next turned at evaluating whether LIF can protect CCA cells from the cytotoxic effects of chemotherapeutic drugs currently used in the treatment of CCA. HuCCT-1 and TFK-1 cells were treated with CDDP, GEM, and GEM+CDDP after a pre-incubation with/without LIF, and their viability assessed by MTS. A drug-induced decrease in cell viability by 34–89% and 23–64% was observed in HuCCT-1 and TFK-1 cells, respectively (Table 2). This cytotoxic effect was significantly counteracted by rhLIF, which augmented cell viability by up to 69.0 ± 36.7% in HuCCT-1 (Figure 3A) and 73.1 ± 17.7% in TFK-1 (Figure 3B) cells. To understand whether cytoprotection was related to anti-apoptotic mechanisms promoted by LIF, we assessed active caspases 3/7. Up-regulation of these caspases is the hallmark of an apoptotic response and is observed in CCA cell lines exposed to GEM+CDDP. The pre-treatment with rhLIF significantly reduced caspases 3/7 activity by 24% in HuCCT-1 and 22% in TFK-1 cells compared to cells challenged with GEM+CDDP in absence of LIF pre-treatment (Figure 3C, 3D). LIF's ability to exert cytoprotective effects in CCA was confirmed in cells silenced for LIFR. Following treatment with GEM+CDDP, genetic inactivation of LIFR in cells exposed to rhLIF led to an increased activation of caspases 3/7 of an extent comparable to scrambled cells and to cells without rhLIF pre-treatment (Figure 3C, 3D).

**Table 2:** Percentage of viable CCA cells following a 24-hour treatment with chemotherapeutic drugs normalized to untreated cells (MTS assay)

	GEM (30 μM)	CDDP (17 μM)	Mix
**HuCCT-1**	38.32 ± 2.40	65.66 ± 4.10	10.87 ± 1.07
**TFK-1**	62.03 ± 7.21	77.16 ± 4.54	35.79 ± 5.36

GEM, gemcitabine; CDDP, cisplatin; Mix, GEM + CDDP

### LIF did not induce a stem cell-like phenotype in established CCA cell lines

To evaluate whether the protective potential of LIF against apoptosis was associated with a dedifferentiation of CCA cells to a stem cell-like phenotype, as described for LIF in malignant melanoma [17], we studied the gene expression of stem cell markers, Nanog and Oct4, following LIF stimulation. However, no significant up-regulation of either stem cell marker was detected in LIF-treated compared to untreated cells (Supplementary Figure 5A, 5B).

### LIF-induced Mcl-1 up-regulation is mediated by PI3K/Akt, but neither by STAT3 nor ERK1/2 activation, in CCA cells

**Figure 4:**
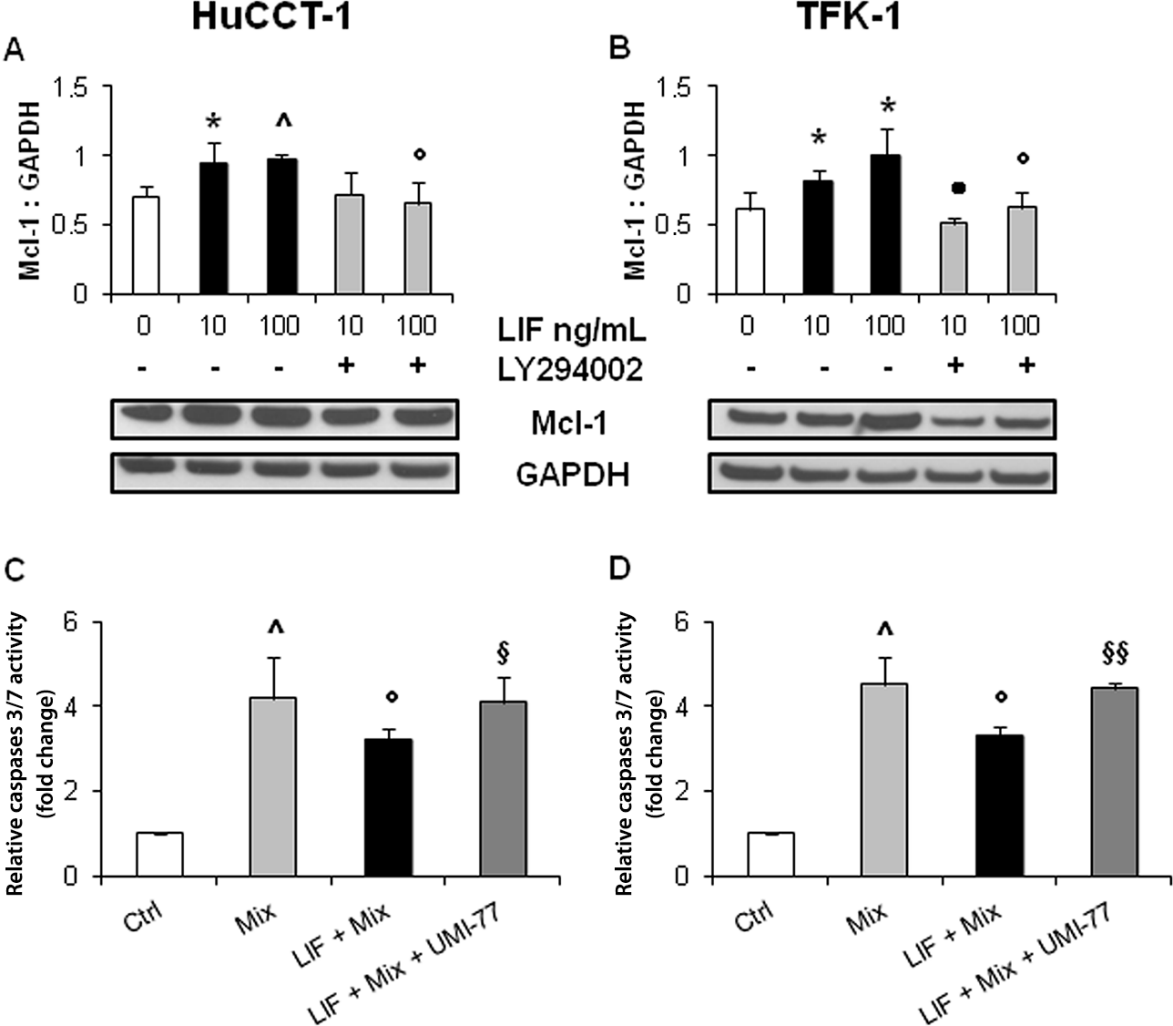
rhLIF's regulation of the anti-apoptotic protein Mcl-1, and caspases 3/7 activity via Mcl-1, in CCA cells By WB, rhLIF induced a significant up-regulation of Mcl-1 (anti-apoptotic) (**A, B.** black columns), in both HuCCT-1 A. and TFK-1 B. cells compared with untreated cells; this effect was abrogated by the specific PI3K inhibitor, LY294002 (A, B gray columns). Representative blots are shown below each respective graph. Interestingly, the protective effect of rhLIF pre-treatment from GEM+CDDP (Mix) cytotoxicity (**C, D.** black columns) was abolished by UMI-77, a selective, small molecule inhibitor of Mcl-1 (C, D dark gray columns) (*p < 0.05 vs. untreated; ^*p* < 0.01 vs. untreated; •*p* < 0.01 vs. LIF 10 without LY294002; °*p* < 0.05 vs. LIF 100; ^§^*p* < 0.05 vs Mix treatment; ^§§^*p* < 0.01 vs Mix treatment; *n* = minimum of 3).

**Figure 5:**
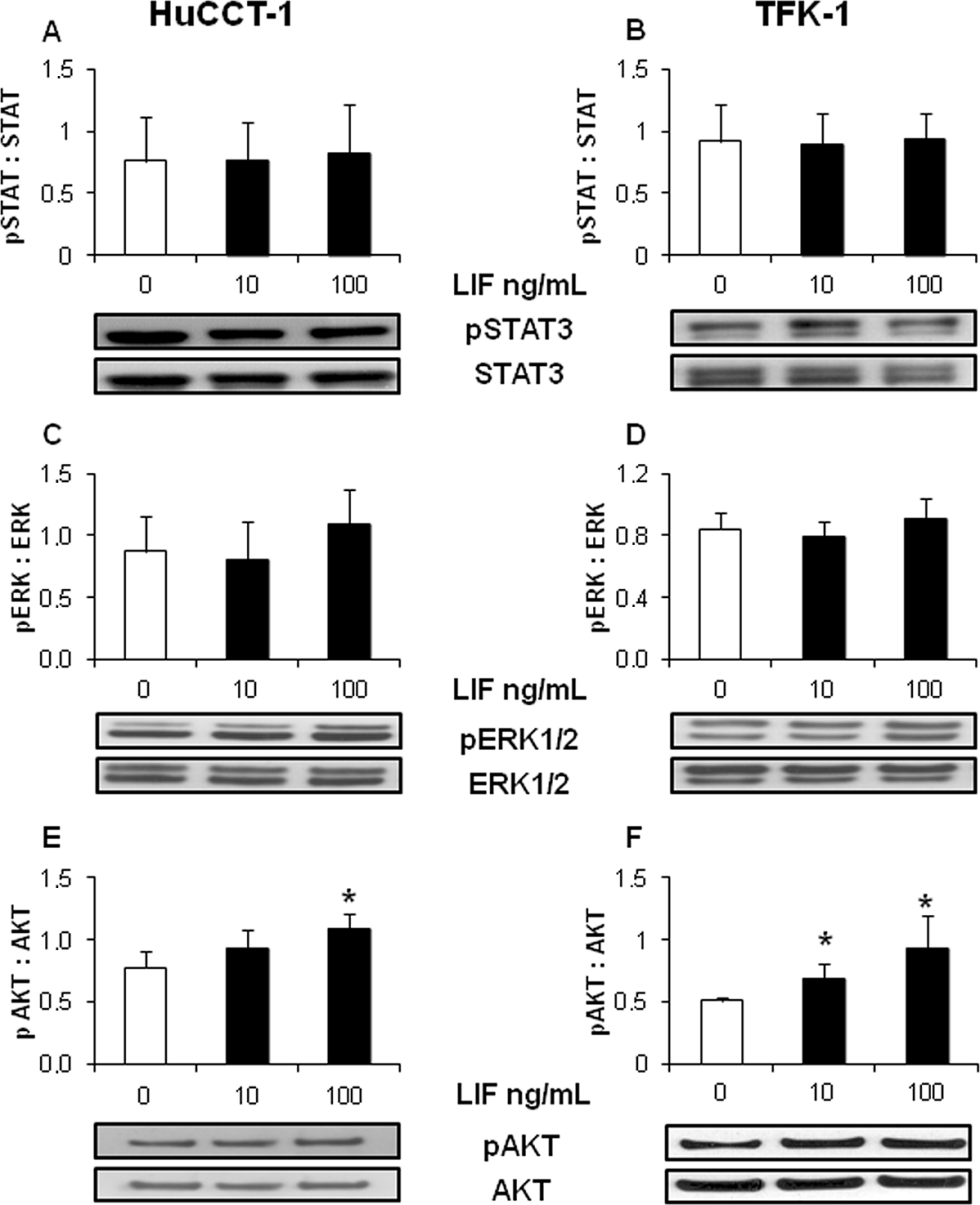
rhLIF acts through a STAT3/ERK1/2-independent, AKT-dependent pathway Whereas rhLIF did not modify the phosphorylation levels of STAT3 **A. B.**, or ERK1/2 **C. D.** in either HuCCT-1 or TFK-1 cell lines by WB, it did induce a significant phosphorylation of AKT **E. F.** compared with untreated cells. Representative blots are shown below each respective graph (*p < 0.05 vs. untreated; *n* = minimum of 3).

To dissect the intracellular signaling that mediates the protective effect of LIF against drug-induced apoptosis in CCA cells, relative expression levels of pBax (pro-apoptotic protein), and Bcl-2 and Mcl-1 (anti-apoptotic proteins) were studied by WB in LIF pre-treated HuCCT-1 and TFK-1 cells. Compared with untreated cells, pBax levels remained unchanged (Supplementary Figure 6A, 6B), whilst Mcl-1 levels increased significantly in both CCA cell lines (Figure 4A, 4B). In contrast with pBax and Mcl-1, Bcl-2 was not expressed in either HuCCT-1 or TFK-1 cells (data not shown). LIF did not induce any significant changes in the phosphorylation levels of STAT3 (Figure 5A, 5B), an effector classically activated by LIF [21], or of ERK1/2 (Figure 5C, 5D) in either CCA cell line. In contrast, LIF stimulated the phosphorylation of AKT (Figure 5E, 5F). The modulatory effect of the PI3K/AKT pathway on LIF-induced Mcl-1 up-regulation was confirmed by treating CCA cells with LY294002, a specific PI3K inhibitor, which significantly reduced Mcl-1 expression in LIF-stimulated HuCCT-1 and TFK-1 cells (Figure 4C, 4D).

### Mcl-1 inactivation prevented LIF cytoprotective effects from chemotherapy-induced apoptosis in CCA cells

To demonstrate that Mcl-1 plays a central role in the LIF-dependent protection of CCA cells from the cytotoxic effects of chemotherapeutic drugs, we evaluated drug-induced apoptosis in HuCCT-1 and TFK-1 cells treated with the selective Mcl-1 inhibitor UMI-77. At our given dosage (10 μM) [22], UMI-77 did not induce any change in cell viability (data not shown), nor affect Mcl-1 expression in both cell lines (data not shown). By caspase 3/7 activation assay, apoptosis of GEM+CDDP-treated cells exposed to UMI-77 and LIF was comparable to GEM+CDDP-treated cells without LIF (Figure 4C, 4D). Together, these data point towards an Mcl-1-mediated anti-apoptotic effect of LIF against chemotherapeutic drug toxicity in CCA, which occurs in a PI3K/AKT-dependent, STAT3- and MAPK-independent manner.

## DISCUSSION

In this study we investigated the role of LIF in CCA, a malignancy with extremely poor prognosis, with a view to unveiling molecular mechanisms responsible for its peculiar aggressiveness, and that may be amenable of therapeutic intervention. We demonstrate that in CCA: 1) LIF is expressed both in the bile ducts, particularly in the ‘ductular-like’ rather than ‘mucin-producing’ subtype, and the stromal cell compartment, including CAF and TAM; 2) its cognate receptor LIFR is selectively up-regulated in neoplastic cholangiocytes; 3) LIF primarily aids tumoral cholangiocytes to resist apoptosis induced by the chemotherapeutic agents GEM and CDDP, without affecting cell proliferation, invasion or the gain of stemness signatures; 4) anti-apoptotic mechanisms are mediated by Mcl-1, through activation of the PI3K/AKT pathway without involving the conventional LIF downstream effector STAT3 or MAPK/ERK; 5) *in vitro* inactivation of Mcl-1 prevents cytoprotective effects exerted by LIF from GEM+CDDP-induced apoptosis in CCA cells.

In CCA, both cholangiocytes and stromal cells (CAF and inflammatory cells) are a source of LIF; conversely, LIF was not expressed by bile ducts in the peritumoral regions. In tumoral epithelia, LIF expression was heterogeneous, with a greater extensiveness in areas of a ductular phenotype. These findings are consistent with a recent study showing that LIF is overexpressed in CCA in conjunction with Oncostatin M, another IL-6 family cytokine that is closely related to LIF, with pleiotropic functions in cell differentiation, proliferation and invasion [23]. In culture conditions, we could confirm that LIF was variably secreted by CCA cells, in keeping with the heterogeneous distribution observed in histological specimens. LIF production by tumor cells directly correlates with a more invasive phenotype [24].

LIF secretion may be induced by several pro-inflammatory cytokines, such as TNF-α, IL-6, IL-1β and TGF-β, variably released by macrophages and activated T-cells populating the local inflammatory microenvironment [12, 13, 25], as well as by the hypoxic conditions typically featured in CCA [17]. Once secreted, LIF itself may induce the expression of LIFR by malignant cells, thus stimulating a positive loop [12, 17]. Our immunohistochemical, WB and real-time PCR data show that LIFR was selectively up-regulated in CCA bile ducts. Transduction of LIF signaling occurs through its binding to the LIF receptor complex consisting of LIFR and the glycoprotein gp130. As opposed to LIFR, every cell type within the human body can express gp130 [26]. Nevertheless, we verified the expression of gp130 and found that it displayed a profile similar to LIFR, thereby indicating that LIF signaling is functionally active in tumoral cholangiocytes. Therefore, the *de novo* expression of LIF and the up-regulation of LIFR in CCA bile ducts, along with LIF overexpression in the tumor reactive stroma, indicate the presence of autocrine and paracrine LIF-mediated mechanisms in CCA. Znoyko et al. also suggested that an autocrine LIF/LIFR axis is active in reactive ductules of cirrhotic livers based on their intense neoexpression compared with bile ducts in normal liver, likely acting as an important signal for ductular reaction [27]. It is interesting to note that in our CCA series, LIF expression was more prevalent in the tumoral areas characterized by a ‘ductular-like’ appearance than by a ‘mucin-producing’ phenotype. These two specific iCCA phenotypes have been recently proposed to originate from topographically distinct cholangiocyte subpopulations, the major hilar ducts for the ‘mucin-producing’ form, and the smaller ducts associated with hepatic progenitor cells for the ‘ductular-like’ variant [28]. Further studies are warranted to understand whether LIF expression may indeed represent a signature of an iCCA subtype arising from hepatic progenitor cells.

Despite the observation that LIF was prevalently expressed in the ‘ductular-like’ areas of CCA, we found that LIF did not exert any proliferative or pro-invasive effects in tumoral cholangiocytes. LIF's effects on neoplastic cells are highly variable, and its failure to stimulate proliferation or invasiveness has also been reported in other epithelial cancers [20]. The nature of the functional effect of LIF in different cancer cell types are dependent upon the signal transduction pathways that may be activated downstream of the receptor [11].

Our data illustrate that LIF enabled CCA cells to resist the pro-apoptotic effects induced by chemotherapeutic agents, such as GEM and CDDP, recently proposed in the treatment of advanced CCA [6]. In CCA cells that were exposed to GEM+CDDP treatment, LIF was able to increase their viability by up to 73% compared with the LIF-untreated cells. In accordance with these findings, LIF was able to hamper the increase of active caspases 3/7 induced by GEM+CDDP by 22–24% in both CCA cells, a fundamental step initiating the cascade of events ultimately leading to apoptosis. Relevance of LIF signaling in conferring anti-apoptotic properties to CCA cells was confirmed by the restoration of cytotoxicity from GEM+CDDP when LIFR was silenced.

To study the mechanisms of resistance to drug-induced apoptosis mediated by LIF, we first evaluated the possible involvement of LIF in inducing a stem cell-like phenotype in CCA cells. Cancer stem cells have an unlimited capacity for self-renewal and a high capacity for drug resistance, and therefore their activation in CCA may explain the failure of current chemotherapies. LIF was recently reported for regulating stemness transcription factors, including Nanog and Oct4, in malignant melanoma [17]. Furthermore, Nanog and Oct4 are recognized as signatures of a stem cell-like phenotype in multiple types of human cancer as well as molecular players of resistance to gemcitabine or cisplatin treatment [29, 30]. However, in our experimental conditions, LIF failed to influence their gene expression levels, meaning the anti-apoptotic effect of LIF is unlikely to be related to a dedifferentiation of CCA cells to a cancer stem cell phenotype. Therefore, we turned to study the balance between the pro-apoptotic and anti-apoptotic proteins of the Bcl-2 family. The anti-apoptotic Bcl-2 family member Mcl-1 acts as a survival factor both in hematogenous and solid tumors, and is currently regarded as a major oncogene [31]; its presence has been reported in both normal and malignant cholangiocytes [32]. We demonstrated that Mcl-1 expression is further augmented by LIF treatment in CCA cells, whilst the pro-apoptotic pBax expression remained unchanged, suggesting that this dysregulation could be a pivotal mechanism responsible for the resistance to apoptosis induced by LIF. The JAK/STAT3, MAPK/ERK or PI3K pathways are three effectors putatively activated by LIF signaling [15, 16, 33] and may be involved in the regulation of Mcl-1 expression in the IL-6-mediated resistance to apoptosis in CCA [9, 10, 33]. Therefore, we first looked at the activation of STAT3 and ERK1/2, classical signals downstream of LIF [11, 33]. However, LIF was unable to alter the levels of either pSTAT3 or pERK1/2 in CCA cholangiocytes, *in vitro*. On the other hand, LIF stimulation up-regulated pAKT in both CCA cell lines, while their treatment with LY294002, a specific PI3K inhibitor, reduced the LIF induced up-regulation of Mcl-1. This demonstrates that the positive modulation of Mcl-1 in CCA cells is dependent upon PI3K/AKT activation, as reported in breast cancer [16], nasopharyngeal carcinoma [18] and rhabdomyosarcoma cells [34]. The chemoresistant effects of LIF have also been reported in colorectal cancer cells by negatively regulating the tumor-suppressor p53 through a STAT3-dependent pathway [35]. In our *in vitro* model, Mcl-1 inactivation by UMI-77 restored sensitivity of CCA cells challenged with LIF to chemotherapeutic agents. This finding is in accordance with recent data indicating that maritoclax, a similar selective inhibitor of Mcl-1 via stimulation of its proteosomal degradation, is able to potently enhance drug-induced apoptosis exerted by the small-molecule Bcl-2 inhibitor ABT-737 in melanoma cells [36].

**Figure 6:**
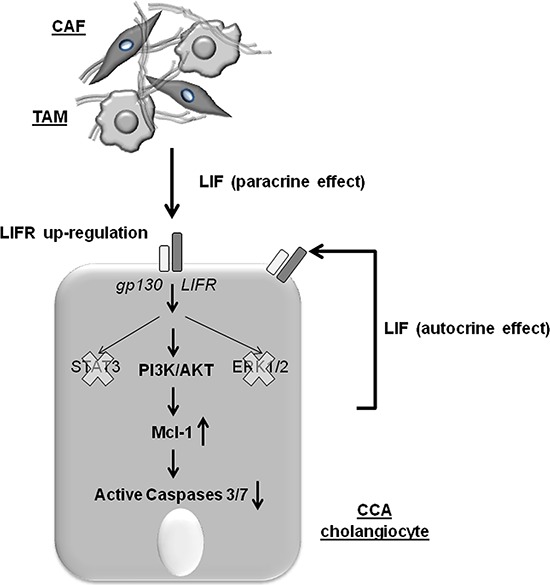
The working model illustrating the molecular mechanisms underlying the chemotherapy-resisting effects of LIF in neoplastic cholangiocytes In CCA, LIFR is up-regulated by cholangiocytes under the influence of LIF released by both the neoplastic cholangiocytes (autocrine loop) and the tumor reactive stromal cells, including CAF and TAM (paracrine loop). When LIFR dimerizes with gp130, LIF signaling is transduced through PI3K/AKT rather than the conventional STAT3 or MAPK/ERK pathways to increase levels of the anti-apoptotic protein Mcl-1, which confers resistance to the cytotoxic effects of chemotherapeutic agents by reducing activation of caspases 3/7.

Overall, our results indicate that LIF signaling in CCA is a mechanism that promotes cancer growth and progression. Whereas LIF does not affect cell proliferation or invasion of cancer cells, it protects malignant cholangiocytes from chemotherapy-induced apoptosis via a STAT3- and MAPK-independent, PI3K/AKT-dependent Mcl-1 activation. The pro-oncogenic effects of LIF rely on its secretion by both the tumoral cells themselves and the adjacent reactive stromal cells acting on the LIFR aberrantly expressed by neoplastic bile ducts (Figure 6). In particular, the autocrine effect is prominent in the ‘ductular-like’ areas of iCCA. On the other hand, LIF-mediated paracrine effects highlight the treatment-resistant functions exerted by the tumor reactive stroma in epithelial cancers with abundant desmoplasia. Since our histological samples were obtained from patients undergoing surgical resection, and, in our center, chemotherapy is only reserved for those with advanced CCA (which generally do not perform histological evaluation), correlating LIF/LIFR expression with clinical data was not possible in the present study. However, we demonstrated that the downstream effectors of LIF signaling may represent innovative molecular targets amenable to therapeutic modulation to increase CCA responsiveness to conventional chemotherapy.

## MATERIALS AND METHODS

### Tissue samples

Formalin-fixed, paraffin-embedded sections of surgically resected CCA liver from 19 patients were included in the immunohistochemical study and compared with the corresponding peritumoral areas where available (*n* = 12). The patients were predominantly male (12/19), with a median age of 64 years (min 35; max 81), and 63% (12/19) were iCCA. CCA areas were then categorized as ‘ductular-like’ or ‘mucin-producing’ according to Komuta [28].

### Cell lines

Three established human CCA cell lines were used: EGI-1, TFK-1 (both eCCA, purchased from Deutsche Sammlung von Mikroorganismen und Zellkulturen, DSMZ, Germany), and HuCCT-1 (iCCA, from Health Science Research Resource Bank, HSRRB, Japan), along with primary biliary cell preparations obtained from surgically resected human iCCA liver samples (*n* = 7), as described [37]. Human cholangiocytes isolated from liver explants of alcoholic liver cirrhosis (*n* = 2) served as controls. All specimens were reviewed by the same dedicated pathologist (AF) to confirm diagnosis. Local regional ethical committee approval was obtained for tissue collection and cell preparations.

### LIF, LIFR and gp130 expression in tissues

By immunohistochemistry we evaluated the expression of LIF, LIFR and gp130 in bile ducts and the stromal compartment in both neoplastic and matched peritumoral areas. Further details are provided in the supplementary online section. The extent of immunoreactivity was scored by two independent observers (SDM, MC) as: 0 = < 5%; 1 = 5–30%; 2 = 30–70%; 3 = > 70% area of positive cells, as reported [38]. In selected tissue specimens, dual immunofluorescence for LIF and α-SMA (myofibroblast marker) or CD45 (inflammatory cell marker) was performed to assess the specific contribution of the different stromal cell types to LIF production.

### LIF and LIFR expression in cells

To evaluate conformity of immunohistochemical findings with *in vitro* data, LIF and LIFR expression were then assessed in cultured cholangiocytes by immunocytochemistry. Further details are provided in the supplementary online section. LIFR protein expression was also assessed by WB in both established and primary CCA cholangiocyte lines. See Supplementary Materials for details. Furthermore, gene expressions of LIF and LIFR on CCA cell lines were evaluated by real-time PCR (see Supplementary Materials).

### LIF secretion by cultured cholangiocytes

The supernatants of CCA and control cholangiocytes cultured for 24 h at a density of 5 × 10^4^ were evaluated for the presence of secreted LIF using an ELISA kit, according to the supplier's instructions (Raybiotech, Milan). For each experiment a LIF standard curve was generated.

### Cell proliferation

HuCCT-1 and TFK-1 cells were cultured at a density of 1 × 10^4^ for 48 h with/without exposure to increasing doses (0.1, 1, 10, 100 ng/mL) of recombinant human LIF (rhLIF, R&DSystems). Proliferation activity was assessed by MTS assay according to the supplier's instructions (CellTiter 96 AQueous One Solution Cell Proliferation Assay, Promega).

### Cell viability

MTS assay was also used to assess whether LIF (24 h pre-treatment at 0.1, 1, 10, 100 ng/mL) affected viability of HuCCT-1 and TFK-1 cells in response to a 24 h treatment with cisplatin, 17 μM (CDDP; Sigma-Aldrich) [39] and gemcitabine, 30 μM (GEM; Sigma-Aldrich) [40], either alone or in combination (GEM+CDDP).

### Stem cell-like phenotyping

Real-time PCR was used to assess LIF effects (100 ng/mL) on Nanog and Oct4 gene expression. RNA was isolated from cultured cells, as described [41]. Further details are available in the supplementary section.

### Cell invasiveness

The invasiveness of HuCCT-1 and TFK-1 cells was assessed by Boyden chamber assay as described [42]. Methodology is detailed in the Supplementary Materials.

### Downstream effectors of LIF signaling in CCA cells

After exposure of HuCCT-1 and TFK-1 cells to rhLIF (10, 100 ng/mL) for 15 mins (STAT3, pSTAT3, ERK1/2, pERK1/2, AKT, pAKT) or 24 h (Bax, pBax, Bcl-2 and Mcl-1), their expression levels were evaluated by WB (see Supplementary Materials). To unravel the pathway regulating Mcl-1, its protein expression was also measured in CCA cells treated with the PI3K chemical inhibitor, LY294002 (10 μM, Sigma) [43], for 10 mins, then with rhLIF plus inhibitor for 24 h.

### Activation of caspases 3/7

HuCCT-1 and TFK-1 cells were seeded into a 96-well plate at 1 × 10^4^ per well with/without rhLIF (100 ng/mL) for 24 h followed by treatment with GEM+CDDP for 12 h. The luminescence-based solution Caspase-Glo 3/7 (Promega) was then used to assess activation of caspases 3/7. Luciferase reaction was evaluated using a microplate reader (BMG Labtech).

### Mcl-1 inactivation

Apoptotic response following GEM+CDDP treatment, assessed as described above (Activation of caspases 3/7), was also evaluated upon Mcl-1 inhibition in HuCCT-1 and TFK-1 cells. We used a novel, selective, small molecule inhibitor of Mcl-1, UMI-77 (10 μM), for 24 h [22]. Antagonism of Mcl-1 function by UMI-77 does not depend on the down-regulation of this protein, but on the ability to block the heterodimerization of Mcl-1 with several members of the Bcl-2 family, including Bax, Bak and Noxa [22, 44]. Inhibitory effect of UMI-77 is related to its binding to the BH3-binding groove of Mcl-1.

### Silencing of LIFR

Gene silencing was performed using commercially available siRNAs against LIFR, and scramble RNA was used as a control (Life Technologies). HuCCT-1 and TFK-1 cell lines were transfected using Lipofectamine 2000 transfection reagent (Life Technologies). Further details are provided in the supplementary section.

### Statistical analyses

Results are shown as the mean ± standard deviation. Statistical comparisons were made using Student's *t*-test. Statistical analyses were performed using SPSS 20.0 software (IBM Corp.). A *p* value < 0.05 was considered significant.

## SUPPLEMENTARY MATERIAL AND METHODS



## Abbreviations

CCA: cholangiocarcinoma
eCCA: extrahepatic CCA
iCCA: intrahepatic CCA
CAF: cancer-associated fibroblasts
TAM: tumor-associated macrophages
IL: interleukin
TNF: tumor-necrosis factor
LIF: leukemia inhibitory factor
LIFR: leukemia inhibitory factor receptor
gp: glycoprotein
JAK: Janus kinase
STAT: signal transducer and activator of transcription
MAPK: mitogen-activated protein kinase
ERK: extracellular signal-regulated kinase
PI3K: phosphatidylinositol-3 kinase
PBS: phosphate-buffered saline
HRP: horseradish peroxidase
TBS: tris-buffered saline
Bcl-2: B-cell lymphoma-2
Mcl-1: myeloid cell leukemia 1
pSTAT: phosphorylated STAT
CC3: cleaved caspase 3
rhLIF: recombinant human LIF
CDDP: cisplatin
GEM: gemcitabine

## References

[1] Bragazzi M, Cardinale V, Carpino G, Venere R, Semeraro R, Gentile R, Gaudio E, Alvaro D (2011). Cholangiocarcinoma: Epidemiology and risk factors. Transl Gastrointest Cancer.

[2] Razumilava N, Gores GJ (2013). Classification, Diagnosis, and Management of Cholangiocarcinoma. Clin Gastroenterol Hepatol.

[3] Bridgewater J, Galle PR, Khan SA, Llovet JM, Park JW, Patel T, Pawlik TM, Gores GJ (2014). Guidelines for the diagnosis and management of intrahepatic cholangiocarcinoma. J Hepatol.

[4] Quyn AJ, Ziyaie D, Polignano FM, Tait IS (2009). Photodynamic therapy is associated with an improvement in survival in patients with irresectable hilar cholangiocarcinoma. HPB (Oxford).

[5] Gatto M, Bragazzi MC, Semeraro R, Napoli C, Gentile R, Torrice A, Gaudio E, Alvaro D (2010). Cholangiocarcinoma: update and future perspectives. Dig Liver Dis.

[6] Valle J, Wasan H, Palmer DH, Cunningham D, Anthoney A, Maraveyas A, Madhusudan S, Iveson T, Hughes S, Pereira SP, Roughton M (2010). Cisplatin plus gemcitabine versus gemcitabine for biliary tract cancer. N Engl J Med.

[7] Rizvi S, Gores GJ (2013). Pathogenesis, diagnosis, and management of cholangiocarcinoma. Gastroenterology.

[8] Zabron A, Edwards RJ, Khan SA (2013). The challenge of cholangiocarcinoma: dissecting the molecular mechanisms of an insidious cancer. Dis Model Mech.

[9] Meng F, Yamagiwa Y, Ueno Y, Patel T (2006). Over-expression of interleukin-6 enhances cell survival and transformed cell growth in human malignant cholangiocytes. J Hepatol.

[10] Kobayashi S, Werneburg NW, Bronk SF, Kaufmann SH, Gores GJ (2005). Interleukin-6 contributes to Mcl-1 up-regulation and TRAIL resistance via an Akt-signaling pathway in cholangiocarcinoma cells. Gastroenterology.

[11] Mathieu ME, Saucourt C, Mournetas V, Gauthereau X, Theze N, Praloran V, Thiébaud P, Bœuf H (2012). LIF-dependent signaling: new pieces in the Lego. Stem cell reviews.

[12] Kamohara H, Ogawa M, Ishiko T, Sakamoto K, Baba H (2007). Leukemia inhibitory factor functions as a growth factor in pancreas carcinoma cells: Involvement of regulation of LIF and its receptor expression. Int J Oncol.

[13] McKenzie RC, Szepietowski J (2004). Cutaneous leukemia inhibitory factor and its potential role in the development of skin tumors. Dermatol Surg.

[14] Gadient RA, Patterson PH (1999). Leukemia inhibitory factor, Interleukin 6, and other cytokines using the GP130 transducing receptor: roles in inflammation and injury. Stem cells.

[15] Zouein FA, Kurdi M, Booz GW (2013). LIF and the heart: just another brick in the wall?. Eur Cytokine Netw.

[16] Li X, Yang Q, Yu H, Wu L, Zhao Y, Zhang C, Yue X, Liu Z, Wu H, Haffty BG, Feng Z, Hu W (2014). LIF promotes tumorigenesis and metastasis of breast cancer through the AKT-mTOR pathway. Oncotarget.

[17] Kuphal S, Wallner S, Bosserhoff AK (2013). Impact of LIF expression in malignant melanoma. Exp Mol Pathol.

[18] Liu SC, Tsang NM, Chiang WC, Chang KP, Hsueh C, Liang Y, Juang JL, Chow KP, Chang YS (2013). Leukemia inhibitory factor promotes nasopharyngeal carcinoma progression and radioresistance. J Clin Invest.

[19] Kellokumpu-Lehtinen P, Talpaz M, Harris D, Van Q, Kurzrock R, Estrov Z (1996). Leukemia-inhibitory factor stimulates breast, kidney and prostate cancer cell proliferation by paracrine and autocrine pathways. Int J Cancer.

[20] Kamohara H, Sakamoto K, Ishiko T, Masuda Y, Abe T, Ogawa M (1997). Leukemia inhibitory factor induces apoptosis and proliferation of human carcinoma cells through different oncogene pathways. Int J Cancer.

[21] Heinrich PC, Behrmann I, Haan S, Hermanns HM, Muller-Newen G, Schaper F (2003). Principles of interleukin (IL)-6-type cytokine signaling and its regulation. The Biochem J.

[22] Abulwerdi F, Liao C, Liu M, Azmi AS, Aboukameel A, Mady AS, Gulappa T, Cierpicki T, Owens S, Zhang T, Sun D, Stuckey JA, Mohammad RM (2014). A novel small-molecule inhibitor of mcl-1 blocks pancreatic cancer growth *in vitro* and *in vivo*. Mol Cancer Ther.

[23] Kang MJ, Kim J, Jang JY, Park T, Lee KB, Kim SW (2014). 22q11-q13 as a hot spot for prediction of disease-free survival in bile duct cancer: integrative analysis of copy number variations. Cancer Genet.

[24] Clark EA, Golub TR, Lander ES, Hynes RO (2000). Genomic analysis of metastasis reveals an essential role for RhoC. Nature.

[25] Albrengues J, Bourget I, Pons C, Butet V, Hofman P, Tartare-Deckert S, Feral CC, Meneguzzi G, Gaggioli C (2014). LIF mediates proinvasive activation of stromal fibroblasts in cancer. Cell Rep.

[26] Wolf J, Rose-John S, Garbers C (2014). Interleukin-6 and its receptors: A highly regulated and dynamic system. Cytokine.

[27] Znoyko I, Sohara N, Spicer SS, Trojanowska M, Reuben A (2005). Expression of oncostatin M and its receptors in normal and cirrhotic human liver. J Hepatol.

[28] Komuta M, Govaere O, Vandecaveye V, Akiba J, Van Steenbergen W, Verslype C, Laleman W, Pirenne J, Aerts R, Yano H, Nevens F, Topal B, Roskams T (2012). Histological diversity in cholangiocellular carcinoma reflects the different cholangiocyte phenotypes. Hepatology.

[29] Lu Y, Zhu H, Shan H, Lu J, Chang X, Li X, Lu J, Fan X, Zhu S, Wang Y, Guo Q, Wang L, Huang Y (2013). Knockdown of Oct4 and Nanog expression inhibits the stemness of pancreatic cancer cells. Cancer Lett.

[30] Noh KH, Kim BW, Song KH, Cho H, Lee YH, Kim JH, Chung JY, Kim JH, Hewitt SM, Seong SY, Mao CP, Wu TC, Kim TW (2012). Nanog signaling in cancer promotes stem-like phenotype and immune evasion. J Clin Invest.

[31] Beroukhim R, Mermel CH, Porter D, Wei G, Raychaudhuri S, Donovan J (2010). The landscape of somatic copy-number alteration across human cancers. Nature.

[32] Okaro AC, Deery AR, Hutchins RR, Davidson BR (2001). The expression of antiapoptotic proteins Bcl-2, Bcl-X(L), and Mcl-1 in benign, dysplastic, and malignant biliary epithelium. J Clin Pathol.

[33] Isomoto H, Kobayashi S, Werneburg NW, Bronk SF, Guicciardi ME, Frank DA, Gores GJ (2005). Interleukin 6 upregulates myeloid cell leukemia-1 expression through a STAT3 pathway in cholangiocarcinoma cells. Hepatology.

[34] Wysoczynski M, Miekus K, Jankowski K, Wanzeck J, Bertolone S, Janowska-Wieczorek A, Ratajczak J, Ratajczak MZ (2007). Leukemia inhibitory factor: a newly identified metastatic factor in rhabdomyosarcomas. Cancer Res.

[35] Yu H, Yue X, Zhao Y, Li X, Wu L, Zhang C, Liu Z, Lin K, Xu-Monette ZY, Young KH, Liu J, Shen Z, Feng Z (2014). LIF negatively regulates tumor-suppressor p53 through Stat3/ID1/MDM2 in colorectal cancers. Nat Commun.

[36] Pandey MK, Gowda K, Doi K, Sharma AK, Wang H-G, Amin S (2013). Proteosomal degradation of Mcl-1 by maritoclax induces apoptosis and enhance the efficacy of ABT-737 in melanoma cells. PloS One.

[37] Massani M, Stecca T, Fabris L, Caratozzolo E, Ruffolo C, Furlanetto A, Morton S, Cadamuro M, Strazzabosco M, Bassi N (2013). Isolation and characterization of biliary epithelial and stromal cells from resected human cholangiocarcinoma: a novel *in vitro* model to study tumor-stroma interactions. Oncol Rep.

[38] Fabris L, Cadamuro M, Moserle L, Dziura J, Cong X, Sambado L, Nardo G, Sonzogni A, Colledan M, Furlanetto A, Bassi N, Massani M, Cillo U (2011). Nuclear expression of S100A4 calcium binding protein increases cholangiocarcinoma invasiveness and metastasization. Hepatology.

[39] Chattopadhyay S, Machado-Pinilla R, Manguan-García C, Belda-Iniesta C, Moratilla C, Cejas P, Fresno-Vara JA, de Castro-Carpeño J, Casado E, Nistal M, Gonzalez-Barón M, Perona R (2006). MKP1/CL100 controls tumor growth and sensitivity to cisplatin in non-small-cell lung cancer. Oncogene.

[40] Shord SS, Camp JR, Young LA (2005). Paclitaxel decreases the accumulation of gemcitabine and its metabolites in human leukemia cells and primary cell cultures. Anticancer Res.

[41] Spirli C, Locatelli L, Morell CM, Fiorotto R, Morton SD, Cadamuro M, Fabris L, Strazzabosco M (2013). PKA dependent p-Ser- beta-catenin, a novel signaling defect in a mouse model of Congenital Hepatic Fibrosis. Hepatology.

[42] Cadamuro M, Nardo G, Indraccolo S, Dall'olmo L, Sambado L, Moserle L, Franceschet I, Colledan M, Massani M, Stecca T, Bassi N, Morton S, Spirli C (2013). Platelet-derived growth factor-D and Rho GTPases regulate recruitment of cancer-associated fibroblasts in cholangiocarcinoma. Hepatology.

[43] Spirli C, Okolicsanyi S, Fiorotto R, Fabris L, Cadamuro M, Lecchi S, Tian X, Somlo S, Strazzabosco M (2010). Mammalian target of rapamycin regulates vascular endothelial growth factor-dependent liver cyst growth in polycystin-2-defective mice. Hepatology.

[44] Mohammad RM, Muqbil I, Lowe L, Yedjou C, Hsu HY, Lin LT, Siegelin MD, Fimognari C, Kumar NB, Dou QP, Yang H, Samadi AK, Russo GL (2015). Broad targeting of resistance to apoptosis in cancer. Semin Cancer Biol.

