# Supplementary material for: Leukemia inhibitory factor protects cholangiocarcinoma cells from drug-induced apoptosis via a PI3K/AKT-dependent Mcl-1 activation
Source: Oncotarget. 2015 Jul 20;6(28):26052–64. doi: 10.18632/oncotarget.4482 (PMC4694885; doi:10.18632/oncotarget.4482)
Supplement: Supplementary file 1 [file oncotarget-06-26052-s001.pdf]

SUPPLEMENTARY MATERIAL AND METHODS

Immunohistochemistry and immunofluorescence

For immunohistochemical staining, after deparaffinization, sections were rehydrated in alcohol and endogenous peroxidase activity was quenched with methanol containing 3% H_2O_2 for 30 mins. Antigen retrieval was performed by steam-heating the slides for 20mins in either 10 mmol/L citrate buffer (pH 6) for LIF or 1.27 mM ethylenediaminetetraacetic acid buffer (pH 8) for LIFR and gp130. Blocking was achieved using UltraVision protein block (ThermoScientific) for 8 mins. The sections were incubated with primary antibodies against LIF (1:50, Santa Cruz), LIFR (1:80, Santa Cruz) and gp130 (1:25, Santa Cruz) overnight at 4°C. After rinsing with phosphate-buffered saline 1M (PBS) supplemented with 0.05% Tween20 (PBS-T; both Sigma), slides were incubated for 30 mins at room temperature with EnVision horseradish peroxidase (HRP)-conjugated secondary antibody (DAKO). All antibodies were diluted in PBS. Specimens were developed using 3,3-diaminobenzedine tetrahydrochloride 0.04 mg/mL (Sigma) with H_2O_2 0.01% diluted in PBS and counterstained with Gill's Hematoxylin N°2 (Sigma). Specimens were analyzed with an Eclipse E800 microscope (Nikon) and LuciaG 5.0 software (Nikon), and images were collected with a digital camera (Nikon, DS-U1).

For immunofluorescence, sections were deparaffinized and rehydrated as described above. Antigen retrieval was performed as above using 10 mmol/L citrate buffer (pH 6). Blocking was achieved as above. The sections were incubated with primary antibodies against LIF (1:50, Santa Cruz), and CD45 (1:300, DAKO) or α-SMA (1:100, DAKO) overnight at 4°C. Slides were then incubated for 30 mins at room temperature with the respective secondary Alexa Fluor 488- or 594-conjugated antibody (1:500, Life Technologies) and mounted with Vectastain + DAPI (Vector Laboratories).

Immunocytochemistry

After fixation with 4% paraformaldehyde (Carlo Erba), cells were incubated overnight at 4°C with primary antibodies against LIF (1:100), and LIFR (1:100). After washing with PBS-T, the cells were incubated for 30 mins at room temperature with the appropriate Alexa Fluor 488 secondary antibody (1:500, Life Technologies) and then mounted with Vectastain + DAPI.

ELISA for LIF quantification

Cells were seeded into a 24-well plate at 5×10^4 per well. After 24 h, supernatants were harvested, stored at –80°C and then the ELISA was developed according to the supplier (RayBiotech). A calibration curve was generated for each experiment.

Western blotting (WB)

Equal concentrations of total lysate obtained from cultured cells were electrophoresed on a 4–12% NuPAGE® Novex Bis-Tris gel (Life Technologies) and proteins were transferred to a nitrocellulose membrane (Life Technologies). The membrane was then blocked with 5% non-fat dry milk (Euroclone) in Tris-buffered saline (TBS) supplemented with 0.1% Tween-20 (TBS-T) for 1 hour and then incubated overnight at 4°C with rabbit anti-LIFR (1:1000, Santa Cruz), rabbit anti-Bax (1:500, Santa Cruz), rabbit anti-phosphorylated Bax (pBax) (1:1000; Bioss), rabbit anti-B-cell lymphoma (Bcl)-2 (1:500, Santa Cruz), rabbit anti-myeloid cell leukemia (Mcl)-1 (1:1000, Cell Signaling), rabbit anti-STAT3, rabbit anti-phosphorylated STAT3 (pSTAT3) (both 1:1000, Cell Signaling), rabbit anti-AKT (1:1000, Cell Signaling), rabbit anti-phosphorylated AKT 1/2/3 (pAKT) (1:1000, Santa Cruz), rabbit anti-ERK1/2 (1:1000, Cell Signaling), and rabbit anti-phosphorylated ERK1/2 (pERK1/2) (1:1000, Cell Signaling). As a reference protein, mouse anti-GAPDH (1:10000, Santa Cruz) was used. The membrane was washed 3 times with TBS-T before incubation with goat anti-mouse (1:2000, Sigma) or goat anti-rabbit (1:2000, Bio-Rad) HRP-conjugated secondary antibodies for 1 hour. Proteins were visualized using enhanced chemiluminescence (SuperSignal West Pico, Thermo Scientific).

Stem cell-like phenotyping

Briefly, untreated and rhLIF-treated (100 ng/mL for 24 hours) HuCCT-1 and TFK-1 cells were homogenized in 1 mL TRIzol® Reagent (Life Technologies). Template complementary DNA was obtained by reverse transcription using 0.5 µg of total RNA, Superscript II reverse transcriptase (Life Technologies), random hexamers (50 pmol), and oligo-dT primers (100 pmol) (Promega). Relative transcript levels were quantified using Taqman gene expression probes for human Nanog and Oct4 (Life Technologies) and the real-time PCR was performed on an ABI 7500 thermocycler (Applied Biosystems). The relative expression of each gene was normalized against that of GAPDH.

Gene expression of LIF and LIFR

Using the same approach described for stem-cell phenotyping, we quantified the relative expression levels of LIF and LIFR in established and primary CCA cell

lines using specific Taqman gene expression commercially available probes (Life Technologies).

Cell invasiveness

Briefly, 5×10^4 cells were re-suspended in serum-free medium and seeded over a polyvinylpyrrolidone-free polycarbonate, 8 µm-pore membrane (Transwell, Costar) coated with 50 µg/mL Matrigel, within a Boyden microchamber. The lower chambers contained serum-free medium with/without rhLIF (10, 100 ng/mL). After 48 hours, cells on the upper surface of the membrane were removed with a cotton swab whilst cells that adhered to the lower surface were fixed and stained using a Diff-Quick Staining Set (Medion Diagnostics); ten random fields of each membrane were photographed to count the number of clearly discernible nuclei.

Silencing of LIFR

Gene silencing of LIFR was performed using commercially available siRNAs against LIFR; scramble RNA served as a control (both Life Technologies). HuCCT-1 and TFK-1 cell lines were transfected using 20 pM of siRNA and Lipofectamine 2000 transfection reagent (Life Technologies) 24 h after plating. Transfection efficiency was assessed by WB and real-time PCR for LIFR.

SUPPLEMENTARY FIGURES

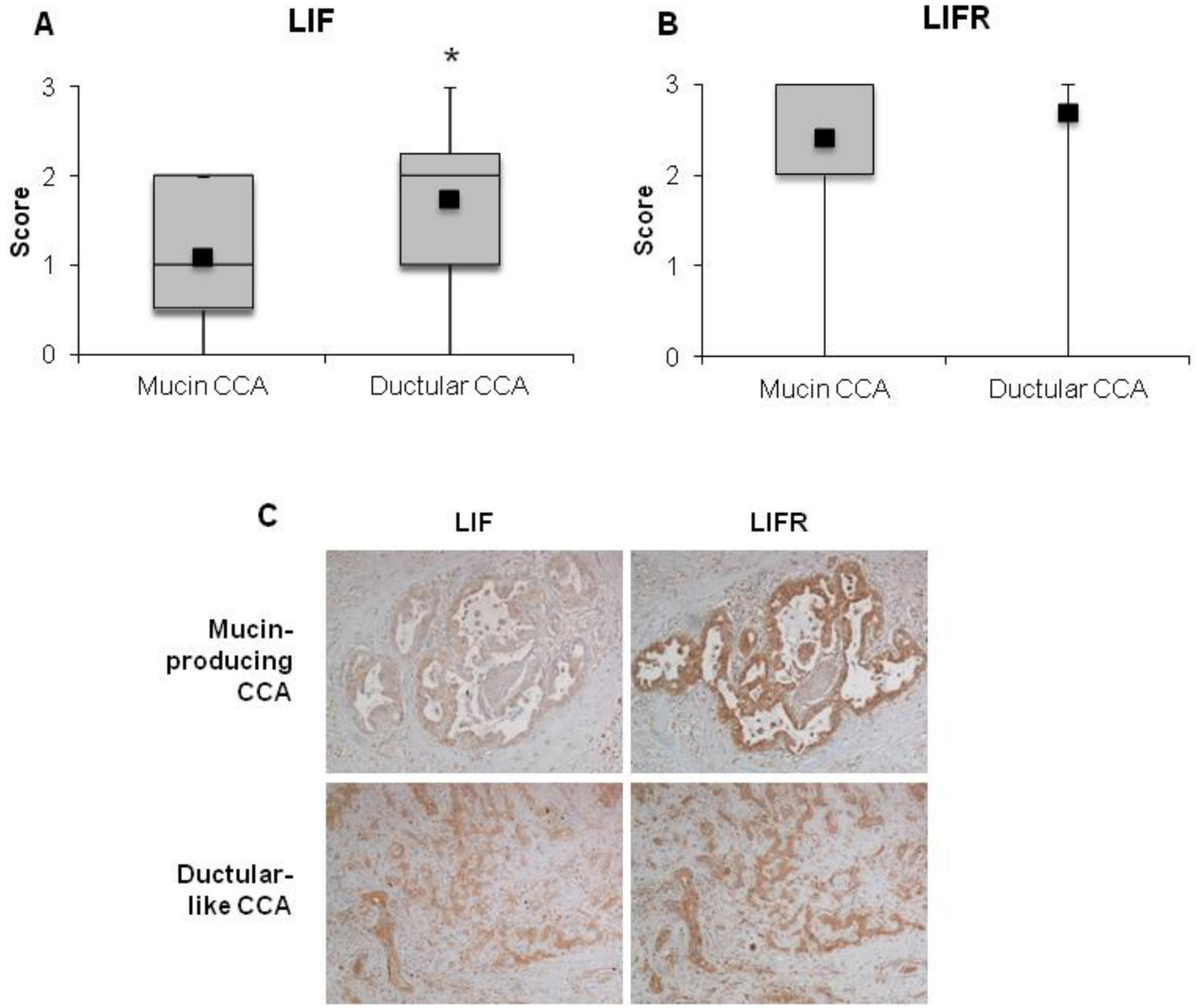

Supplementary Figure 1: Extensiveness of LIF and LIFR expression in 'ductular-like' and 'mucin-producing' areas of CCA. LIF expression was more extensive in 'ductular-like' than in 'mucin-producing' CCA bile ducts **A.**, whereas no significant differences in LIFR staining were observed between the two CCA phenotypes **B.** Representative micrographs illustrating LIF and LIFR staining in sequential sections of 'mucin-producing' (upper panels) and 'ductular-like' (lower panels) CCA areas are shown **C.** (Original magnification: 200 x; *$p < 0.05$).

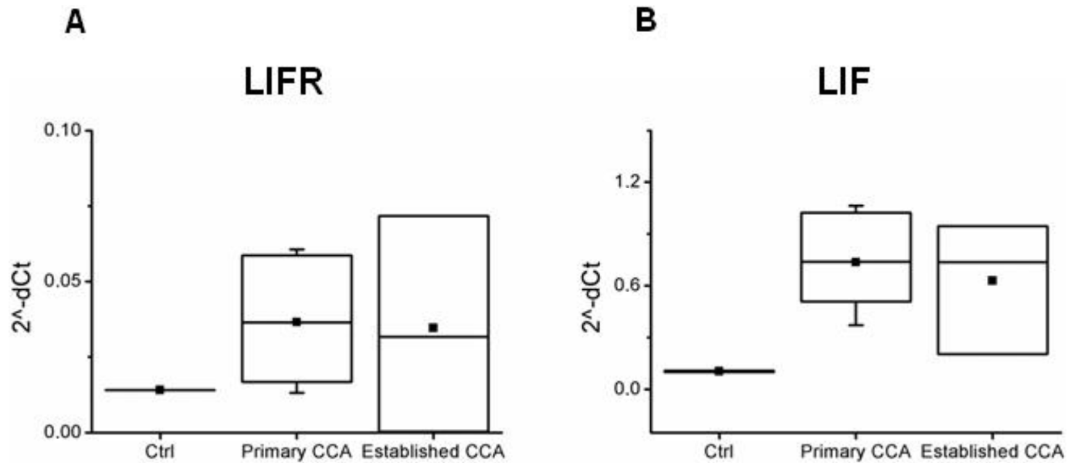

Supplementary Figure 2: LIFR and LIF gene expression quantification in human primary control and CCA cholangiocytes, and established CCA cell lines. To confirm data obtained by WB and ELISA, the gene expressions of LIFR **A.** and LIF **B.** were assessed in primary (n = 7) and established (n = 3) CCA cell lines, and in control (n = 2) cholangiocytes by real-time PCR. The results show increased mRNA levels for both receptor and ligand in CCA cells compared with controls.

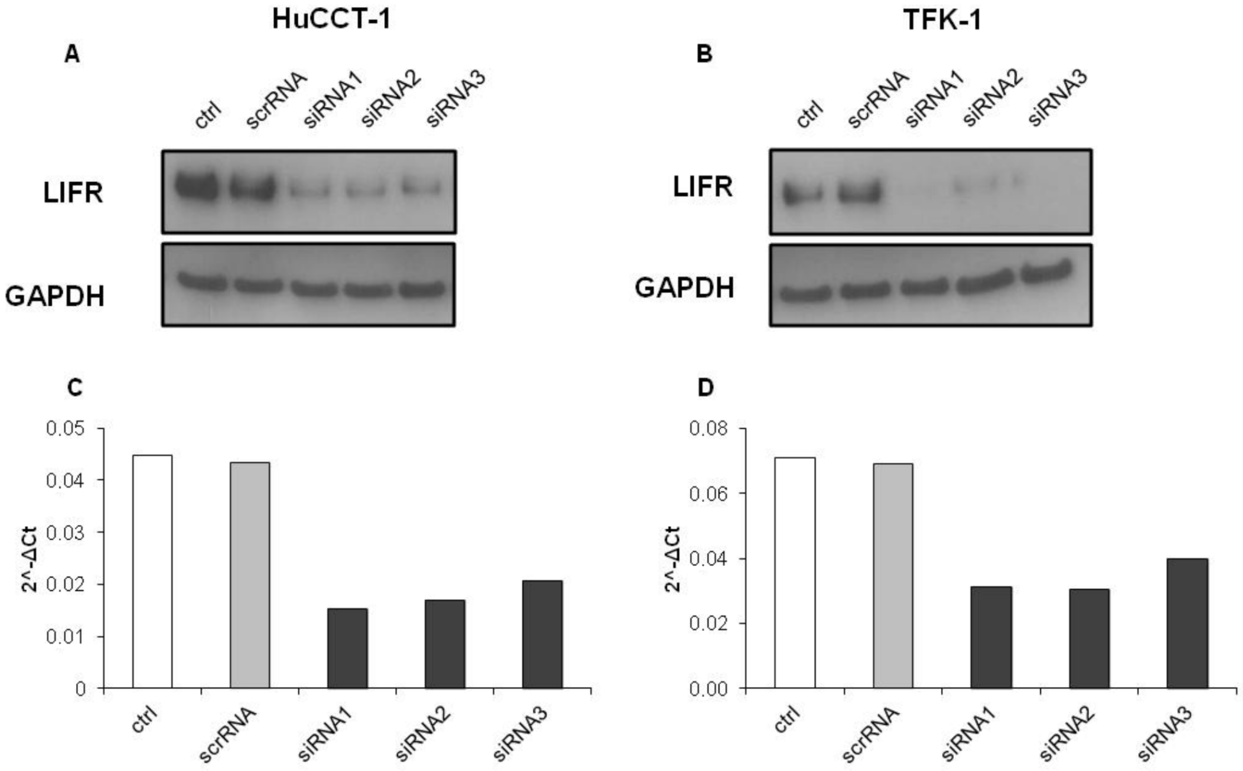

Supplementary Figure 3: Effects of siRNA on LIFR expression in CCA cells. The ability of three different siRNAs (siRNA1, siRNA2, and siRNA3) to suppress LIFR expression was evaluated by both WB and real-time PCR in HuCCT-1 **A. C.** and TFK-1 **B. D.** cells. All siRNAs induced a pronounced reduction in protein (A, B) and transcript levels (C, D) of LIFR. Therefore, siRNA1 and siRNA2 were selected for the experiments on cell proliferation (shown in Supplementary Figure 4C, 4D) and drug-induced cytotoxicity (shown in Figure 3C, 3D).

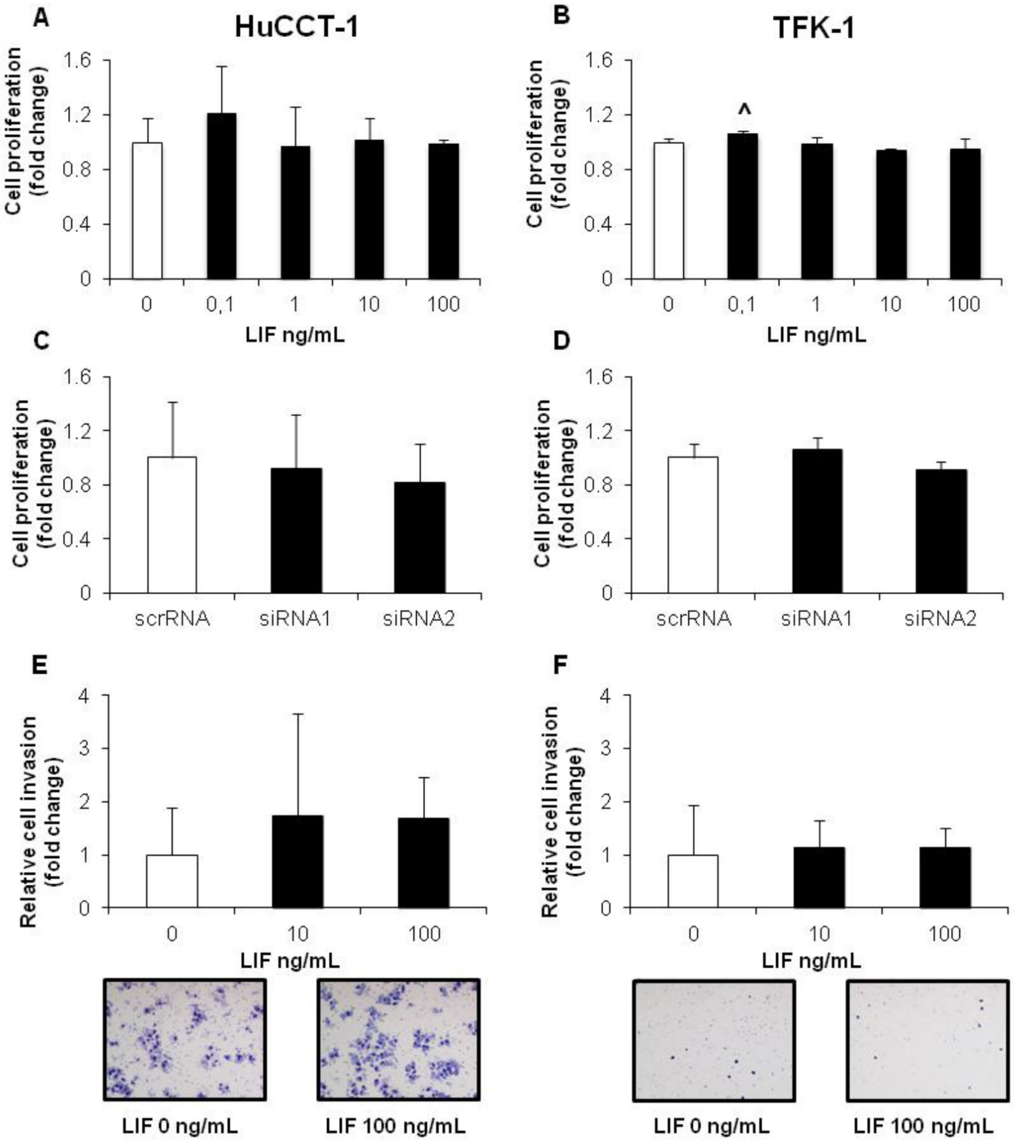

Supplementary Figure 4: LIF did not affect cell proliferation and invasion of HuCCT-1 and TFK-1 cells. By MTS assay, HuCCT-1 **A.** and TFK-1 **B.** cells challenged with rhLIF at increasing doses, did not show any relevant changes in cell proliferation, except for a minimal increase with the lowest dose in TFK-1 (^$p < 0.01$ vs. LIF 0; $n = 3$ in duplicate). Interestingly, in absence of rhLIF stimulation, LIFR silencing did not reduce cell proliferation of HuCCT-1 **C.** and TFK-1 **D.** cells compared with scramble, thus ruling out a possible constitutive activation of cell proliferation that could be exerted by autocrine LIF. Additionally, no effects were observed on cell invasion with either HuCCT-1 **E.** or TFK-1 **F.** cells upon LIF stimulation, as assessed in Boyden chamber assays (n = minimum of 3). Micrographs of representative fields of invaded cells in response to medium alone or LIF supplementation are illustrated (Original magnification: 100 ×).

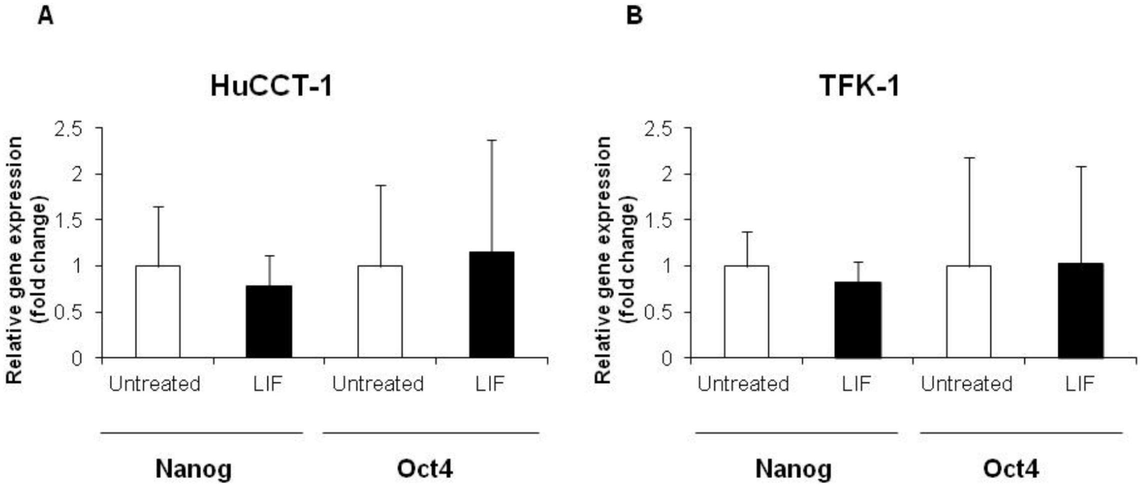

Supplementary Figure 5: rhLIF did not increase gene expression of Nanog and Oct4 in HuCCT-1 and TFK-1 cells. By real-time PCR, rhLIF stimulation (100 ng/mL) was unable to change Nanog and Oct4 mRNA levels in HuCCT-1 **A.** and TFK-1 **B.** cells (n = 3).

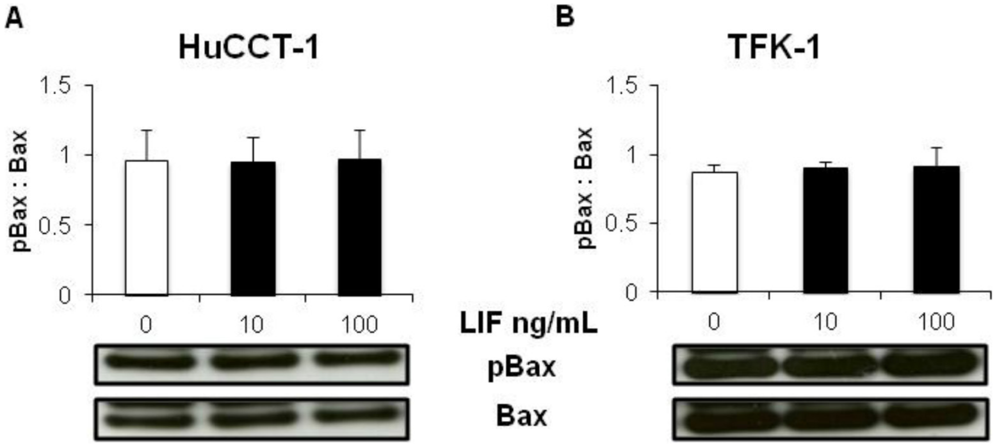

Supplementary Figure 6: Effects of rhLIF on Bax phosphorylation in CCA cells. By WB, rhLIF did not modify pBax:Bax (pro-apoptotic) in either HuCCT-1 **A.** or TFK-1 **B.** cells compared with untreated cells. Representative blots are shown below each respective graph n = 3).
